# Ketogenic diet: Possible mechanism, old and new applications

**DOI:** 10.1113/EP090858

**Published:** 2023-01-17

**Authors:** Roberto Cannataro

**Affiliations:** ^1^ Galascreen Laboratories University of Calabria Rende Cosenza Italy; ^2^ Research Division Dynamical Business & Science Society – DBSS International SAS Bogotá Colombia

## 1 INTRODUCTION

The ketogenic diet (KD) is now >100 years old; in this period, the attention of the medical profession has fluctuated. Originally intended for a single therapeutic purpose (to manage the forms of infantile epilepsies refractory to drugs), recently its applications have increased, often inappropriately (Wheless, 2008). In the last 20 years, this dietary approach has received renewed scientific interest because it has been applied in support of therapy in various pathologies. It is worth remembering that a KD is a nutritional programme that includes a daily intake of carbohydrates of <30 g or that represents <5% of the total kilocalories, with the rest being derived from protein and fats in variable ratios.

## 2 BIOCHEMISTRY OF KETOGENIC DIET

The KD, like absolute fasting, triggers the synthesis of ketone bodies. The acetone is rapidly expired, whereas the aceto‐acetic acid is rapidly converted into β‐hydroxybutyrate, which is used by the cells for energy. Synthesis of ketone bodies occurs in the liver, in particular, to compensate for the deficiency of glucose for the neurons in the CNS. β‐Hydroxybutyrate can cross the blood–brain barrier (Zhu et al., 2022) and is a more efficient energy source than glucose, entering the Krebs cycle directly. In my opinion, the sensitivity of the CNS to ketone bodies and the KD is a particularly important feature. Before the mechanism of ketosis is activated, most muscular and hepatic glycogen is depleted; in this situation, the weight of the carbohydrate and the water associated with it is lost. This phenomenon has been and still is exploited to propose this nutritional scheme as the magical formula that makes you lose weight very quickly. Also, for this reason, the medical profession has viewed the KD with distrust.

## 3 KETOGENIC DIET AND INFLAMMATION

Although the mechanism of ketogenesis is well understood (Zhu et al., 2022), its actions and influences on physiological or pathological states are unclear. An anti‐inflammatory effect has been proposed (Ciaffi et al., 2021), in addition to the well‐established property of improving body composition and insulin sensitivity; my co‐workers and I showed positive modulation of the inflammatory state by screening microRNA expression (Cannataro, Perri et al., 2019). This has been confirmed, albeit in rats, also by (Lu et al., 2018), which shows a regulation of the intracellular mediators of inflammation and production of free radicals, namely nuclear factor‐E2‐related factor 2 (Nrf2) and nuclear factor‐κB (NF‐κB) (Zhang et al., 2018). However, it remains to be clarified whether this result is attributable to glycaemic regulation and consequent lower production of advanced glycation end‐products or whether ketones have a specific action.

## 4 NEUROLOGICAL ACTION OF KETOGENIC DIET

As mentioned in the Introduction, the first application of the KD was in epilepsy (Pietrzak et al., 2022). Now it is also being applied successfully to recurrent migraine (Di Lorenzo et al., 2021). The mechanism could be shared, and a ‘normalization’ of energy metabolism is hypothesized, which would avoid the excitatory glycaemic spikes (Masino & Rho, 2019); another hypothesis involves direct action on the synthesis and transport of GABA (Zhang et al., 2018). Another application of the KD potentially relates to its ability to manage lipoedema, but the field requires more research. At present, there are only two studies (Cannataro et al., 2021; Sørlie et al., 2022) that prove its effectiveness, including management of pain, often present in this pathological state, although the mechanism remains elusive.

## 5 KETOGENIC DIET AND CANCER AND POSSIBLE OTHER NEW APPLICATIONS

The KD has also been considered in the treatment of cancer. Many cancer cells depend exclusively on glucose, meaning that the KD would effectively starve them (Warburg effect); this, associated with the anti‐inflammatory effect, might provide protective benefit (Bandera‐Merchan et al., 2020; Sørlie et al., 2022; Weber et al., 2020), Currently explored in animal trials (Lu et al., 2018). Finally, KD also shows an epigenetic action. As already shown, the regulation of microRNAs has a considerable impact on protein synthesis, particularly on the enzymes linked to energy metabolism, inflammation and antioxidant status (Cannataro, Caroleo, et al., 2019; Cannataro, Perri et al., 2019). Alternatively, animal findings have indicated an inhibitory effect on DNA‐methyltransferase, probably mediated by the adenosine receptor, and an indirect action on sirtuins, and these mechanisms could have enormous potential (Ungaro et al., 2022). Thus, we are probably only at the beginning of knowledge on the mechanism of the KD, because there is evidence that the KD can have a therapeutic or supportive action or even modulate pathological states, such as sarcopenia (Cannataro et al., 2021).

However, the mechanisms should be clarified by more human studies, not only of efficacy but also to elucidate the mechanisms underlying the efficacy of the KD. To target the structure of the diet (e.g., the ratio between proteins and fats, ranging from 1:1 to 1:6), the duration (compliance is not always optimal) and the daily amount of carbohydrate (even only on some days) could be adjusted according to the physical activity practised. Focusing on the goal, disease or condition that is to be managed, the dietary set‐up could be varied.

## AUTHOR CONTRIBUTIONS

Roberto Cannataro conceived the idea, wrote the first draft of the manuscript, and edited and revised the manuscript. Roberto Cannataro approved the final version submitted for publication and agreed to be accountable for all aspects of the work in ensuring that questions related to the accuracy or integrity of any part of the work are appropriately investigated and resolved. The person designated as author qualifies for authorship, and all those who qualify for authorship are listed.

## CONFLICT OF INTEREST

None declared.

## FUNDING INFORMATION

None.
